# Cervical selective nerve root injection alleviates chronic refractory pain after brachial plexus avulsion: a case report

**DOI:** 10.1186/s40981-022-00574-9

**Published:** 2022-10-12

**Authors:** Yoji Chikama, Aiko Maeda, Ryudo Tanaka, Masachika Tominaga, Kazuhiro Shirozu, Ken Yamaura

**Affiliations:** 1 Department of Anesthesiology and Critical Care Medicine, Kyushu University Hospital, Fukuoka, 812-8582 Japan; 2 Operating Rooms, Kyushu University Hospital, Fukuoka, Japan; 3 Department of Anesthesiology and Critical Care Medicine, Kyushu University Graduate School of Medicine, Fukuoka, Japan

**Keywords:** Brachial plexus avulsion, Ultrasound-guided nerve block, Cervical selective nerve root injection, Chronic pain

## Abstract

**Background:**

Intractable chronic pain, as well as motor, sensory, and autonomic neuropathy, significantly reduces the quality of life of brachial plexus avulsion (BPA) patients. We report the successful application of cervical selective nerve root injection (CSNRI) in a patient with BPA.

**Case presentation:**

A 40-year-old man had been diagnosed with complete left BPA due to a motorcycle accident and underwent intercostal nerve transplantation at the age of 18 years and had been experiencing pain ever since. His pain increased after fracture of the left humerus, and he was referred to our pain management clinic. As his exacerbated pain was suspected to be due to peripheral nerve hypersensitivity, we performed repetitive ultrasound-guided CSNRI (3 mL of 1% mepivacaine of each) targeted C5 and 6 intervertebral foramina, and his symptoms gradually improved.

**Conclusions:**

Repetitive CSNRI may help diagnose and treat BPA-associated peripheral neuropathic pain, even in patients diagnosed with BPA.

## Background

Brachial plexus avulsion (BPA), defined as the preganglionic disruption of the nerve roots from the spinal cord, is a specific type of brachial plexus traction injury (BPI) classified by Parry et al. [1, 2]. Chronic pain after BPA, as well as motor, sensory, and autonomic neuropathy, significantly reduces patients’ quality of life. More than 70% of patients with BPI develop chronic pain, particularly those with BPA, who develop severe refractory chronic pain that resists conventional conservative treatment [3]. This is because BPA-induced pain is thought to be more frequently associated with central neuropathic mechanisms [2, 4]. Little is known about the preclinical and clinical evidence of peripheral neuropathic mechanisms of pain with BPA. Here, we report the long-term effects of repetitive ultrasound-guided cervical selective nerve root injection (CSNRI) for refractory pain in a patient with complete BPA.

This article adheres to the applicable guidelines on enhancing the quality and transparency of health research. Written informed consent was obtained from the patient for publication of the case report.

## Case presentation

A healthy man had a severe motorcycle accident at the age of 18 years and was transported to the hospital. He fell onto his left shoulder during the accident and was diagnosed with a left irreparable complete BPA. The patient underwent nerve transfer from the intercostal nerve to the musculocutaneous nerve for elbow flexion reconstruction. Although the flexion of his left elbow returned to a certain degree after surgery, all other motor paralysis of the left upper extremity and his severe left upper extremity pain symptoms persisted. He was prescribed various medications, including antiepileptics, antidepressants, and weak opioids; however, they did not sufficiently relieve his pain.

At the age of 40 years, he fell down the stairs and fractured his left humerus and subsequently underwent osteosynthesis. Although he did not feel any pain at the time of the fracture, his daily life was impeded by increased pain in his left upper extremity. Two years after the fracture, he was referred to our pain management clinic with the chief complaint of severe pain in the left upper extremities.

**Fig. 1:**
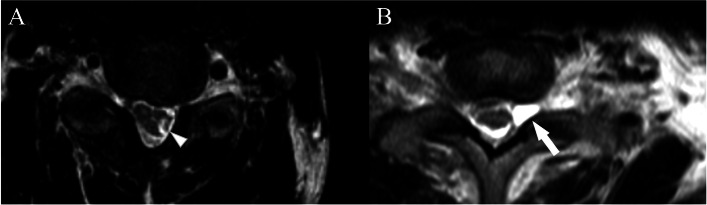
Cervical spine axial T2-weighted magnetic resonance image. **A** High-intensity lesion on the left spinal cord at the level of C5/6 (white arrowhead). **B** High-intensity lesions of C 7/Th1 level intervertebral foramen, considered to be a pseudomeningocele (white arrow)

His maximum and minimum pain scores were 10 and 7, respectively, on a numerical rating scale (NRS; 0–10). He experienced spontaneous, sharp electrical intermittent pain in his left upper extremity that lasted for approximately 10 min and recurred 10–15 times daily. Pain severely interfered with the patient’s sleep and quality of life (QOL) and negatively impacted him at work. Muscle atrophy and edematous changes were observed in the left upper extremities. The manual muscle test result of the upper left extremity was 0/5, and he could not flex the left elbow. He had complete paralysis and loss of sensation in his left upper extremity, and pain occurred throughout his upper left extremity, centered on his upper arm. T2-weighted magnetic resonance imaging (MRI) showed a high-intensity lesion on the left side of C5–6 in the spinal cord and high-intensity lesions (cystic pseudomeningocele) on the left lateral recess of C7-Th1 (Fig. 1). As his pain was exacerbated after the humerus fracture, we determined that CSNRI was a good candidate for diagnosing and treating his pain, which was thought to be peripheral neuropathic pain caused by peripheral nerve hypersensitivity in the C5–6 nerve root innervated area.

**Fig. 2:**
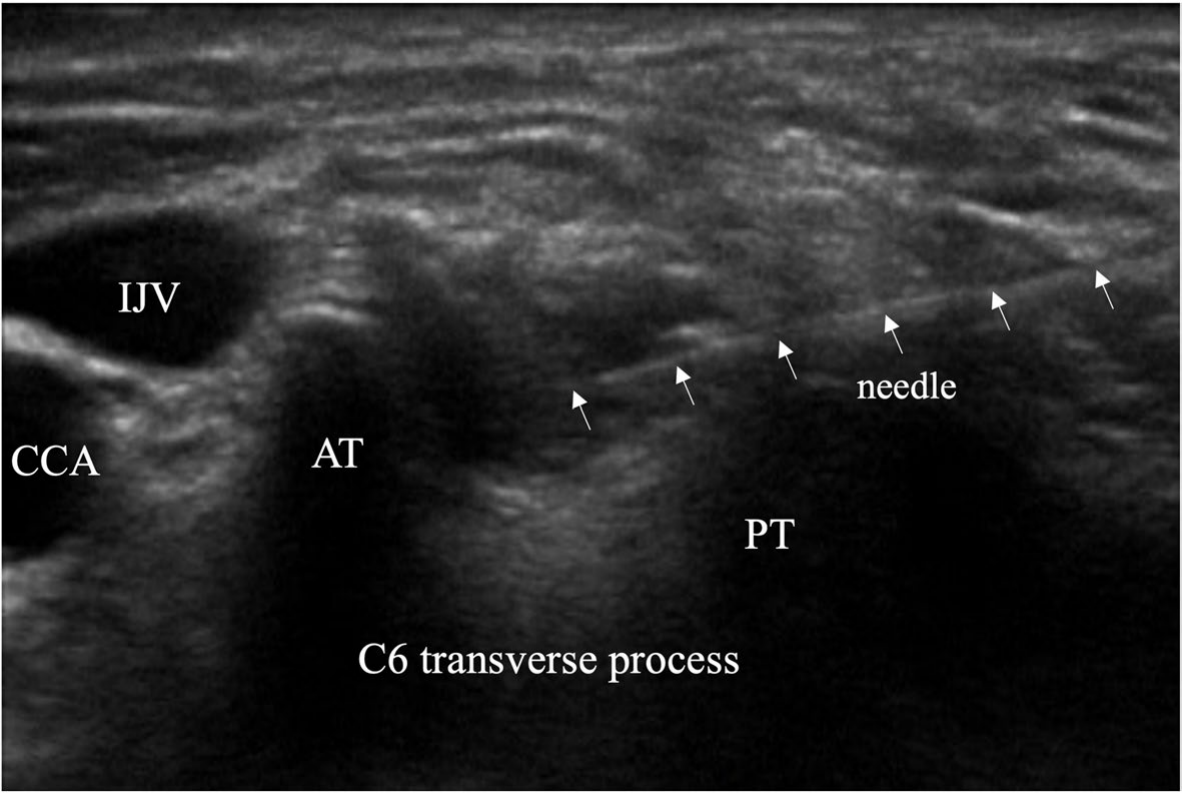
An ultrasound image of cervical selective nerve root injection. A needle (white arrow) is visualized on the right side of the image toward the C6 intervertebral foramina AT, anterior tubercle; CCA, common carotid artery; IJV, internal jugular vein; PT, posterior tubercle

When C5 and 6 intervertebral foramina were confirmed by ultrasound images, each nerve root could not be visually observed. Although not visually evident, we believed that there were nerve roots with slight connections and performed a nerve block using CSNRI at the C5 and 6 intervertebral foramina. The ultrasound image is shown in Fig. 2. A needle (25G injection needle, 60 mm; Top Corporation, Tokyo, Japan) was introduced using in-plane technology. Immediately after injection of 3 mL of 1% mepivacaine, his pain improved significantly. CSNRI reduced his pain; therefore, he received repetitive CSNRI injections about once a week, which reduced his maximum and minimum pain to NRS 4 and 2 continuously. In addition, the frequency of intermittent paroxysmal pain decreased. After a few months, he was able to control his pain with CSNRI several times per month, and his QOL significantly improved. Three years after his initial visit to our clinic, his pain did not worsen with monthly injections.

## Discussion

The brachial plexus is formed by the cervical nerve (C5–8) and the first thoracic nerve (T1). Complete (from C5 to T1 nerve roots) BPA occurs as a result of falling on the shoulder and subsequent brachial plexus over-traction, such as high-energy motorcycle accidents predominantly seen in young men, and causes severe upper extremity dysfunction [5, 6]. Diagnosis of BPA, especially the differentiation between pre- and postganglionic BPI, is of paramount importance in predicting treatment prospects and functional prognosis. Our patient was diagnosed with complete BPA under direct surgical exploration and the cervical spine MRI images, and he experienced refractory pain and upper extremity dysfunction. Patients with BPA frequently experience neuropathic pain, and there is considerable evidence that central mechanisms are involved [2]. The posterior horn and Lissauer tract (LT) of the spinal cord are the first sites where sensory primary afferents transmit peripheral information to the central nervous system. Nociceptive fibers through the LT terminate in the substantia gelatinosa or deep laminae of the posterior horn, and the signals through them are modulated within these laminae [7]. In BPA, besides the nerve root injury, as the primary afferents of the preganglionic side are avulsed from the spinal cord, it is presumed that the posterior horn and LT are directly disrupted and may induce central neuropathic pain [2].

However, several reports have suggested that even in patients with complete BPA, at least one nerve root has not been completely avulsed, and that the non-avulsed ruptured root may be the cause of pain. Bertelli et al. have suggested that the remaining slightly connected ruptured roots cause refractory pain by demonstrating that diagnostic CT-guided selective nerve root block temporally improves pain in patients with chronic BPA [8]. This showed the existence of a peripheral mechanism of pain in the region of the avulsion, and that the central mechanism is not always the only cause of pain. In our patient, it was considered that his pain was enhanced by augmented peripheral nerve sensitivity with a fracture of the humerus. The significant efficacy of ultrasound-guided C5–6 CSNRI is believed to support the notion that the cause of pain in this patient was predominantly a peripheral rather than a central mechanism.

In general, the treatment of intractable chronic pain due to BPA includes conservative medications and surgical therapy. First, first-line pharmacological therapies, such as tricyclic antidepressants and calcium channel ligands, combined physical and occupational therapy, and psychological support is used as treatment modalities. Opioids are addictive and should therefore be avoided [9, 10]. For patient refractory to conservative measures, neurosurgical options are available, including dorsal root entry zone lesioning (DREZ-tomy) or spinal cord stimulation (SCS) procedures. However, DREZ-tomy for the purpose of destroying lamina I to V and terminating the abnormal hyperactivity of the dorsal horn may result in new deafferentation pain. It has been reported that the long-term success rate was 66% as the pain gradually relapses [11, 12]. Moreover, the effect of SCS on BPA is limited [13]. Although peripheral nerve block treatment for BPA is currently limited in its effect, it is useful for diagnosing whether the cause of pain is peripheral, the procedure is simple, and has fewer adverse events compared to neurosurgical procedures. The effects of local anesthetics have been reported to be more complex, including not only the inhibition of voltage-gated sodium channels but also blockade of ionotropic glutamate receptors and voltage-gated calcium channels. These complex actions are believed to affect the downstream pathways in neurons and are speculated that these are long-term effects of chronic neuropathic pain due to suppression of nerve hypersensitivity [14, 15]. Moreover, it was presumed that CSNRI promoted improvement in our patient’s pain as it increased the direct peripheral blood flow by blocking the efferent sympathetic nerves that accompany the peripheral nerves [16]. Few case reports have shown the efficacy of continuing peripheral nerve blocks or ultrasound-guided CSNRI regularly for refractory BPA pain. Therefore, its utility and indications require further investigation in preclinical and clinical studies.

In conclusion, ultrasound-guided CSNRI relieved BPA-related chronic refractory pain in our patient. When BPA symptoms include a peripheral component, repetition of peripheral nerve blocks may have a notable effect on intractable pain and should be attempted before invasive surgery.

## Data Availability

Not applicable.

## Abbreviations

BPA: Brachial plexus avulsion
BPI: Brachial plexus injury
CSNRI: Cervical selective nerve root injection
DREZ-tomy: Dorsal root entry zone lesioning
LT: Lissauer tract
MRI: Magnetic resonance imaging
NRS: Numerical rating scale
QOL: Quality of life
SCS: Spinal cord stimulation
